# Amplicon Sequencing Reveals Microbial Community Structure and Its Relationships with Environmental Factors in *Macrobrachium nipponense* Aquaculture Ponds

**DOI:** 10.3390/microorganisms14050982

**Published:** 2026-04-27

**Authors:** Wanqi Zhang, Xiaofan Fang, Yuefan Zhang, Yiwei Xiong, Wenyi Zhang, Shubo Jin, Hongtuo Fu, Sufei Jiang, Hui Qiao

**Affiliations:** 1 Wuxi Fisheries College, Nanjing Agricultural University, Wuxi 214081, China; 15333937507@163.com (W.Z.); zhangwy@ffrc.cn (W.Z.); jinsb@ffrc.cn (S.J.); fuht@ffrc.cn (H.F.); 2 Key Laboratory of Mariculture & Stock Enhancement in North China’s Sea, Ministry of Agriculture and Rural Affairs, Dalian Ocean University, Dalian 116023, China; m15304476580@163.com (X.F.); 13377712674@163.com (Y.Z.); 3 Key Laboratory of Freshwater Fisheries and Germplasm Resources Utilization, Ministry of Agriculture and Rural Affairs, Freshwater Fisheries Research Center, Chinese Academy of Fishery Sciences, Wuxi 214081, China; xiongyw@ffrc.cn

**Keywords:** pond-cultured oriental *Macrobrachium nipponense*, ribosomal RNA gene amplicon, microbial community, environmental factors

## Abstract

*Macrobrachium nipponense* is one of the major economic species in freshwater aquaculture in China. As an important component of aquaculture ecosystem, microorganisms participate in key processes such as material cycling and water quality regulation, exerting significant impacts on the cultured organisms. In this study, high-throughput sequencing of the 16S rRNA, 18S rRNA, and ITS regions was employed to comparatively analyze the characteristics of microbial communities before and during the cultivation period, combined with correlation analysis of environmental factors. The results showed that the dominant microbial groups in the prawn pond water were Proteobacteria, Cyanobacteria, Ascomycota, Basidiomycota, Chlorophyta, and Arthropoda. The microbial community structure differed significantly between the pond water during the culture period and the pre-culture external river baseline: manifested as an increase in the relative abundances of Cyanobacteria, Chytridiomycota, and zooplankton, and a decrease in the abundances of Ascomycota, Basidiomycota, and Chlorophyta. Analysis of LEfSe revealed that the low-nitrogen pond was enriched with taxa such as Muribaculaceae; the high-nitrogen pond was enriched with taxa such as *Cyanobium_*PCC-6307; and the control pond was enriched with taxa such as *CL500-29_marine_group*. Functional prediction indicated that heterotrophic metabolism-related functions dominated the microbial communities. The abundance of fungal pathogens was significantly higher in the low-nitrogen group, while potential pathogenic bacteria were enriched in the high-nitrogen group. Ammonia nitrogen is a core environmental factor associated with differences in microbial community structure. The findings of this study can provide theoretical references and data support for water quality optimization and the construction of healthy aquaculture models in freshwater shrimp and crab farming waters.

## 1. Introduction

The oriental river prawn, scientifically termed *Macrobrachium nipponense*, belongs to the Palaemonidae family. This species exhibits an extensive geographical range, being indigenous to several nations including China, Japan, Korea, Vietnam, and Myanmar. Over the past few years, this species has been intentionally introduced to several new regions, including Singapore, the Philippines, Iran, Uzbekistan, and southern parts of Iraq [1,2,3]. Within the realm of economics, *Macrobrachium nipponense* emerges as a highly valuable freshwater prawn species across China, Japan, Korea, and Vietnam. Consumers love it for its tender meat and rich nutrition, such as high-quality protein, unsaturated fatty acids, and various minerals [4]. Additionally, it boasts considerable edible merit and exhibits robust competitiveness in the market. In freshwater aquaculture, *M. nipponense* boasts advantages such as a short growth cycle, strong adaptability, and flexible farming models. This makes it one of the dominant species in Chinese freshwater aquaculture, with farming areas covering more than a dozen provinces and municipalities [5,6]. The scale and production of *M. nipponense* cultivation have been increasing annually, reaching an annual output of 226,392 tons in 2023 [7]. Firstly, cultivating *Macrobrachium nipponense* can yield substantial economic returns for aquaculture practitioners. Furthermore, it significantly contributes to securing a stable supply of aquatic products. Additionally, it serves as a catalyst for rural revitalization and aids in refining the industrial structure of aquaculture. Ultimately, it stands as a pivotal species underpinning the sustainable advancement of China’s freshwater fisheries economy.

As the *M. nipponense* farming industry continues to expand and intensify, some practical problems have gradually emerged. For example, in practical production scenarios, issues such as excessively elevated stocking densities and inadequate feeding management practices are frequently observed. These unreasonable farming practices have directly led to a sharp increase in the organic load of the water bodies. Simultaneously, these factors have also led to disruptions in the equilibrium of vital nutrients, including nitrogen and phosphorus, within the aquatic ecosystem. This, in turn, triggers disturbances in microbial community structure, water quality deterioration, ultimately restricting prawn growth and reducing farming efficiency. Excessive accumulation of inorganic nitrogen not only induces environmental problems like water eutrophication and dissolved oxygen depletion but also increases the risk of disease outbreaks by impairing the metabolic and immune functions of *M. nipponense* [8,9,10]. This series of issues is closely related to structural and functional changes in the waterborne microbial community, particularly the core microbial groups involved in the nitrogen cycle. Microorganisms decompose residual feed and feces, converting organic pollutants into inorganic salts [11,12,13], while phytoplankton absorb nutrients through photosynthesis, helping maintain the stability of the pond ecosystem [14,15].

Although numerous studies have focused on microbial communities in aquaculture environments, most suffer from the following limitations: (i) research subjects are largely confined to a single microbial group (e.g., bacteria or fungi), lacking a systematic integrated analysis of multi-kingdom communities including bacteria, fungi, and microeukaryotes (phytoplankton, protozoa, etc.), which hinders a comprehensive understanding of the synergistic succession patterns of microorganisms in aquaculture ecosystems; (ii) existing studies have mostly focused on average levels of total nitrogen or a single nitrogen form, while the differential response mechanisms of microbial communities under different nitrogen gradients (especially high-nitrogen versus low-nitrogen extremes) remain underexplored, making it difficult to identify the key regulatory thresholds of nitrogen loading on microbial community structure; (iii) research on water microbial communities of the economically important *M. nipponense* is still very limited, particularly lacking systematic tracking of the dynamic succession from pre-culture to culture periods.

To address these research gaps, this study focused on *M. nipponense* culture ponds and adopted an extreme group design (selecting ponds with the highest and lowest total nitrogen concentrations) to systematically compare microbial community dynamics between the pre-culture period (external river background) and the culture period (pond water). High-throughput sequencing of 16S rRNA, 18S rRNA, and ITS genes was employed to jointly analyze, for the first time in *M. nipponense* aquaculture, the multi-kingdom community structure of bacteria, microeukaryotes, and fungi. Functional potentials were predicted using PICRUSt2 and FUNGuild, with a focus on changes in nitrogen-cycling-related metabolic pathways and fungal trophic modes. Simultaneous monitoring was conducted on crucial water quality indicators, namely total nitrogen, ammonia nitrogen, nitrate nitrogen, and nitrite nitrogen. Additionally, multivariate statistical techniques, including redundancy analysis, were employed to uncover the response mechanisms of microbial community structure and functionality to environmental variables across varying nitrogen concentrations. The findings are intended to clarify the succession dynamics and ecological roles of microbial communities throughout the culture process of *Macrobrachium nipponense*, thereby providing a theoretical basis for optimizing water quality and constructing a healthy aquaculture ecosystem through targeted regulation of microbial communities.

## 2. Materials and Methods

### 2.1. Sample Collection and Water Quality Parameter Measurement

This study collected samples during the pre-culture and culture stages at the *Macrobrachium nipponense* aquaculture base in Meijiadu, Wuxi City, Jiangsu Province, China. The culture ponds had an average water depth of approximately 1 m. Prior to stocking, all ponds were drained and dried, leaving no water; therefore, it was not possible to collect pre-culture water samples directly from the experimental ponds. To obtain an environmental background reference for the pre-culture period, water was sampled from the external river that supplied the ponds. Three water samples were collected along the longitudinal river section (upstream, midstream, and downstream) and served as biological replicates for the pre-culture stage. Water samples for the culture stage were collected 30 days after stocking. Six parallel culture ponds (each approximately 5 mu, equivalent to 0.33 hectares) were selected. From each pond, three independent water samples were collected at a depth of 0–50 cm from the water surface at the central cross-section (both ends and the center), serving as biological replicates for that pond. A total of 18 water samples were collected during the culture stage (6 ponds × 3 samples/pond).

After collection, each water sample was split into two separate aliquots. One aliquot was promptly filtered using a 0.22 μm mixed cellulose ester filter membrane (HP-MCE-90–022, Hongpu, Shanghai, China). Subsequently, the filters were transferred into sterile 15 mL centrifuge tubes (LABSELECT, Beijing Labgic Technology Co., Ltd., Beijing, China), rapidly frozen in liquid nitrogen, and then stored at −80 °C to facilitate subsequent extraction of microbial DNA. The other portion (unfiltered) was stored on ice and transported to Suzhou Chishan Technology Co., Ltd. (Suzhou, China) for determination of ammonium nitrogen (NH_4_^+^-N), nitrite nitrogen (NO_2_^−^-N), nitrate nitrogen (NO_3_^−^-N), and total nitrogen (TN) concentrations. Based on the water quality analysis results, the ponds with the highest and lowest total nitrogen concentrations were selected from the six culture ponds. To clarify the differences in microbial communities under varying nitrogen levels, an extreme group design was adopted by selecting ponds with the highest and lowest nitrogen concentrations for subsequent analysis, thereby maximizing the environmental gradient and enhancing the sensitivity of intergroup comparisons. All three water samples from each of these two ponds were included, resulting in six culture-stage water samples. These were combined with the three pre-culture river water samples to form a subset of nine water samples for subsequent high-throughput sequencing analysis of the microbial community. It should be noted that each treatment group contained only one independent sampling unit (pond or river); therefore, the statistical inference power of this study is limited, and all conclusions should be regarded as exploratory observations.

During the culture period, all ponds were stocked with ovigerous female shrimp at a uniform density of 1 kg per mu. Commercial formulated feed was provided once daily. The initial feeding rate was 2% of the estimated total shrimp body weight, with daily adjustments made thereafter based on weather conditions, water quality, and observed feeding activity to ensure satiation without excess residual feed.

### 2.2. DNA Extraction, Amplicon Generation, Library Construction, and Sequencing

Genomic DNA was comprehensively extracted from the filtered membranes utilizing the DNeasy PowerSoil Pro Kit (QIAGEN, Hilden, Germany, catalog number 47014), strictly adhering to the manufacturer’s guidelines. In brief, the filter membrane was introduced into a PowerBead Pro Tube, followed by the addition of 800 µL of Solution CD1. The tube was then firmly positioned horizontally on a Vortex Adapter (QIAGEN, Hilden, Germany, catalog number 13000-V1-24) and subjected to vigorous vortexing at the highest speed setting using a Vortex-Genie 2 (Scientific Industries, Bohemia, NY, USA) for a duration of 10 min to ensure effective cell lysis. Subsequently, the sample underwent centrifugation at 15,000× *g* for 1 min, after which the supernatant was carefully transferred to a fresh tube. Subsequently, 200 µL of Solution CD2 and 600 µL of Solution CD3 were added sequentially, with vortex mixing and centrifugation steps to remove impurities and adjust the salt concentration. The cell lysate was then transferred into an MB Spin Column and subjected to centrifugation at 15,000× *g* for 1 min, facilitating the binding of DNA to the silica membrane. The membrane was washed sequentially with 500 µL of Solution EA and 500 µL of Solution C5 to remove residual proteins and salts. Finally, DNA was eluted by adding 50 µL of Solution C6 (10 mM Tris) to the center of the membrane. Subsequently, the sample underwent another round of centrifugation at 15,000× *g* for 1 min. The concentration of the extracted DNA was determined using a NanoDrop 2000 spectrophotometer (Thermo Scientific, Waltham, MA, USA), while its integrity was evaluated through electrophoresis on a 1.2% agarose gel.

To investigate the composition of bacterial, fungal, and microeukaryotic communities, the V4 region of the 16S rRNA gene, the ITS2 region, and the V4 region of the 18S rRNA gene were selectively amplified. The specific primer pairs employed were as follows: for the 16S V4 region, 515F (5′-GTGYCAGCMGCCGCGGTAA-3′) paired with 806R (5′-GGACTACNVGGGTWTCTAAT-3′); for the ITS2 region, ITS3 (5′-GCATCGATGAAGAACGCAGC-3′) combined with ITS4 (5′-TCCTCCGCTTATTGATATGC-3′); and for the 18S V4 region, 528F (5′-GCGGTAATTCCAGCTCCAA-3′) used alongside 706R (5′-AATCCRAGAATTTCACCTCT-3′). Sample-identifying barcode sequences were appended to the 5′ ends of these primers. PCR amplification was carried out utilizing Pfu high-fidelity DNA polymerase (TransGen Biotech, Beijing, China) within a 25 µL reaction mixture. The thermal cycling protocol comprised an initial denaturation step at 95 °C for 2 min, followed by 25 cycles of denaturation at 95 °C for 30 s, annealing at 55 °C (for 16S and 18S targets) or 53 °C (for ITS targets) for 30 s, and extension at 72 °C for 30 s. A final extension step was performed at 72 °C for 5 min. To ensure the reliability of the results, a negative control (utilizing sterile water in place of the template DNA) was incorporated into each amplification batch. Any samples from batches that exhibited a band in the negative control were omitted from subsequent analyses.

The PCR products underwent purification using magnetic beads (Vazyme VAHTSTM DNA Clean Beads, Nanjing, China), followed by quantification with the Quant-iT PicoGreen dsDNA Assay Kit (Thermo Fisher Scientific, Waltham, MA, USA). Subsequently, the purified and quantified PCR products were combined in equimolar concentrations. Sequencing libraries were then constructed utilizing the Illumina TruSeq Nano DNA LT Library Prep Kit (San Diego, CA, USA), a process that encompassed end-repair, A-tailing, and the ligation of adapters incorporating index sequences, magnetic bead-based size selection to remove adapter dimers, library enrichment PCR, and final fragment selection and purification. The quality of the assembled sequencing libraries was evaluated utilizing the Agilent High Sensitivity DNA Kit (Santa Clara, CA, USA) on an Agilent Bioanalyzer, ensuring the presence of a single, well-defined peak without any adapter contamination. Library concentrations were precisely measured using the Quant-iT PicoGreen dsDNA Assay Kit in conjunction with a Promega QuantiFluor fluorometric system (Madison, WI, USA), with samples required to meet a minimum qualified concentration threshold of ≥2 nM for further processing. Qualified libraries were diluted, pooled in equimolar ratios, denatured into single strands with NaOH, and subjected to paired-end sequencing on an Illumina NovaSeq 6000 platform using the NovaSeq 6000 SP Reagent Kit (500 cycles) (San Diego, CA, USA).

### 2.3. Bioinformatics Analysis

Raw sequencing data were subjected to quality filtering, after which samples were demultiplexed based on sample-specific barcode sequences, and primers and adapters were removed. Subsequent data analyses were conducted utilizing QIIME 2 software (version 2019.4). Sequence denoising was conducted with the DADA2 plugin using the following key parameters: --p-trunc-len-f 240, --p-trunc-len-r 200, and --p-max-ee 2.0, while other parameters were left as default. After undergoing denoising, merging of paired-end reads, and elimination of chimeric sequences, amplicon sequence variants (ASVs) were successfully generated. Singleton ASVs (those with a total abundance of 1 across all samples) were removed. Raw sequencing data were subjected to quality filtering, after which samples were demultiplexed based on sample-specific barcode sequences, and primers and adapters were removed. Subsequent bioinformatics analyses were carried out with QIIME 2 software (version 2019.4). Sequence denoising was conducted with the DADA2 plugin using the following key parameters: --p-trunc-len-f 240, --p-trunc-len-r 200, and --p-max-ee 2.0, while other parameters were left as default. After completing the steps of denoising, merging paired-end reads, and eliminating chimeric sequences, amplicon sequence variants (ASVs) were successfully obtained. Singleton ASVs (those with a total abundance of 1 across all samples) were removed. To assess whether the sequencing depth was sufficient to capture the microbial community diversity, rarefaction curves were generated based on the non-rarefied ASV table (Supplementary Figure S1). Subsequent diversity analyses were performed on a rarefied ASV table, with the rarefaction depth set to 95% of the lowest sequence count among samples.

For taxonomic classification, the 16S rRNA gene sequences were annotated by referencing the SILVA database (Release 132, accessible at http://www.arb-silva.de accessed on 20 September 2025). Similarly, the ITS sequences underwent annotation using the UNITE database (Release 8.0, available at https://unite.ut.ee/ accessed on 20 September 2025). Additionally, the 18S rRNA gene sequences were also annotated utilizing the SILVA database (Release 132, http://www.arb-silva.de accessed on 20 September 2025). The classify-sklearn algorithm in QIIME 2, based on a pre-trained Naive Bayes classifier, was employed for annotation, with a confidence threshold set to 0.7.

Alpha diversity was evaluated using the Chao1 index (richness), Shannon index (diversity), Faith’s phylogenetic diversity (PD) index, and Pielou’s evenness index. Differences in alpha diversity between groups were assessed using the Wilcoxon rank-sum test (for two groups) or the Kruskal–Wallis test (for multiple groups). Subsequently, false discovery rate control was implemented using the Benjamini–Hochberg procedure to adjust for multiple comparisons. Microbial community dissimilarities (beta diversity) were visualized through principal coordinate analysis (PCoA) and non-metric multidimensional scaling (NMDS), employing three distinct distance metrics: Bray–Curtis dissimilarity (composition-based), weighted UniFrac (phylogenetic- and abundance-weighted), and unweighted UniFrac (phylogenetic-only). Statistical differences in microbial community structure among experimental groups were evaluated using permutational multivariate analysis of variance (PERMANOVA), conducted via the adonis function in R with 999 permutations. The threshold for statistical significance was defined as *p* < 0.05.

Differential abundance analysis of microbial taxa across experimental groups was performed using two complementary approaches: Linear Discriminant Analysis Effect Size (LEfSe) and Random Forest classification. For LEfSe analysis, the threshold for linear discriminant analysis (LDA) scores was established at ≥2.0 to identify taxa with statistically significant and biologically relevant differences in relative abundance. The Random Forest algorithm was employed to assess feature importance and validate the discriminatory power of identified biomarkers, with model performance evaluated through out-of-bag error estimation. For random forest analysis, feature importance was evaluated using 10-fold cross-validation, and the top 50 ASVs or genera contributing to group differentiation were selected.

The metabolic capacity of the bacterial community was inferred from 16S rRNA gene sequences using PICRUSt2 (v2.5.2), which predicts functional profiles based on phylogenetic relationships to reference genomes in the IMG database. Under default parameters, the Hidden State Prediction algorithm was used to infer the abundance of KEGG orthologs (KOs) and MetaCyc metabolic pathways for each ASV.

### 2.4. Statistical Analysis

All statistical analyses were performed using R software (v4.3.3) [16]. Differences in environmental factors and alpha diversity indices (including Chao1, Shannon, Simpson, Pielou’s evenness, and Faith’s PD) between groups were compared using one-way analysis of variance (ANOVA). When omnibus statistical tests indicated significant differences, Tukey’s Honest Significant Difference (HSD) test was applied for pairwise group comparisons to control for type I error inflation. For beta diversity analysis, group-wise dissimilarities were evaluated using either Analysis of Similarities (ANOSIM) [17] or Permutational Multivariate Analysis of Variance (PERMANOVA) [18] with Bray–Curtis dissimilarity [19] serving as the primary distance metric for both approaches, Jaccard distance [20], and weighted or unweighted UniFrac distance matrices [21,22], with the number of permutations set to 999. The relationships between microbial community structure and environmental variables were assessed using redundancy analysis (RDA) for direct gradient analysis or Mantel tests for matrix correspondence comparisons, depending on data type and research question. In RDA, species data with small variance between groups were log-transformed to meet analytical assumptions. The Mantel test was used to assess the correlation between the species composition distance matrix (Bray–Curtis distance) [19] and the environmental factor distance matrix (Euclidean distance), with 999 permutations. For differential species analysis (e.g., comparisons of abundance between groups in heatmaps), the Wilcoxon rank-sum test was applied, followed by false discovery rate (FDR) correction using the Benjamini–Hochberg method [23]; adjusted *padj* < 0.05 was considered significant. For all statistical analyses, the significance level was set at *p* < 0.05.

### 2.5. Nucleotide Sequence Accession Numbers

The 16S, 18S, and ITS sequence datasets from the pond aquaculture environment have been deposited in the NCBI Sequence Read Archive (SRA) database (https://submit.ncbi.nlm.nih.gov/subs/sra/ accessed on 20 September 2025): 16S rRNA gene amplicon data can be accessed under accession number PRJNA1415883; 18S rRNA gene amplicon data under PRJNA1415895; ITS rRNA gene amplicon data under PRJNA1415902.

## 3. Results

### 3.1. Environmental Factor Analysis

Environmental parameter measurements for different pond ecosystems are summarized in Table 1. Longitudinal analysis revealed significant temporal shifts in nitrogen dynamics during the aquaculture period. Compared to pre-culture conditions (Group O), post-culture samples exhibited marked increases in total nitrogen (TN), ammonia nitrogen (NH_4_^+^-N), and nitrate nitrogen (NO_3_^−^-N) concentrations. Specifically, pre-culture TN concentrations were significantly lower than those in Groups 4 and 6 (*p* < 0.05), while NH_4_^+^-N levels showed no significant differences across temporal comparisons. For NO_3_^−^-N, pre-culture concentrations differed significantly only from Group 4 (*p* < 0.05), whereas nitrite nitrogen (NO_2_^−^-N) remained comparable between pre-culture and all post-culture groups.

Post-culture group comparisons demonstrated distinct nitrogen profile patterns with significant inter-group variations. TN concentrations ranged from the lowest in Group 3 to significantly elevated levels in Group 6 compared to all other groups (*p* < 0.01). Ammonia nitrogen exhibited a similar pattern, with Group 2 showing the lowest concentration and Group 6 the highest (*p* < 0.001). In contrast, nitrate nitrogen concentrations peaked in Group 4 but showed no significant differences among groups (*p* > 0.05). Nitrite nitrogen levels remained generally low across all groups, with Groups 5 and 6 presenting significantly higher concentrations than other groups (*p* < 0.05), though these two groups showed no mutual difference.

Based on these results, group 3 (lowest total nitrogen) and group 6 (highest total nitrogen) were selected as the low-nitrogen group (group L) and high-nitrogen group (group H), respectively, for further analysis of microbial diversity together with the pre-culture baseline (group O). This extreme group design was intended to maximize the environmental gradient and enhance the sensitivity of comparisons between groups.

### 3.2. Sequencing Data Quality

A total of 613,654 (16S), 661,282 (18S), and 637,993 (ITS) high-quality sequences were obtained from the nine samples. Raw and quality-filtered sequencing read counts for all samples are presented in Supplementary Table S1, showing pre- and post-processing quantification metrics. Rarefaction curves based on non-rarefied ASV tables showed that the observed species counts for all samples plateaued when the sequencing depth exceeded approximately 20,000 reads (Supplementary Figure S1), indicating that the sequencing depth was adequate to cover the major microbial taxa. Good’s coverage values exceeded 0.997 for all samples (Table 2), further supporting the sufficiency of sequencing depth.

### 3.3. Microbial Diversity Analysis

All 16S rRNA, 18S rRNA, and ITS region sequences were clustered into 11,289, 4408, and 7617 operational taxonomic units (OTUs), respectively, with each sample showing varying numbers of clustered OTUs (Table 2). For each sample, three alpha diversity metrics (Chao1, Shannon, and Simpson indices) were computed and summarized in Table 2. Statistical comparison using one-way ANOVA demonstrated that none of these diversity indices exhibited significant inter-group differences across the three experimental groups (*p* > 0.05). The highest values of different alpha diversity indices were observed in the low-nitrogen group (16S) and the high-nitrogen group (18S), respectively.

**Figure 1:**
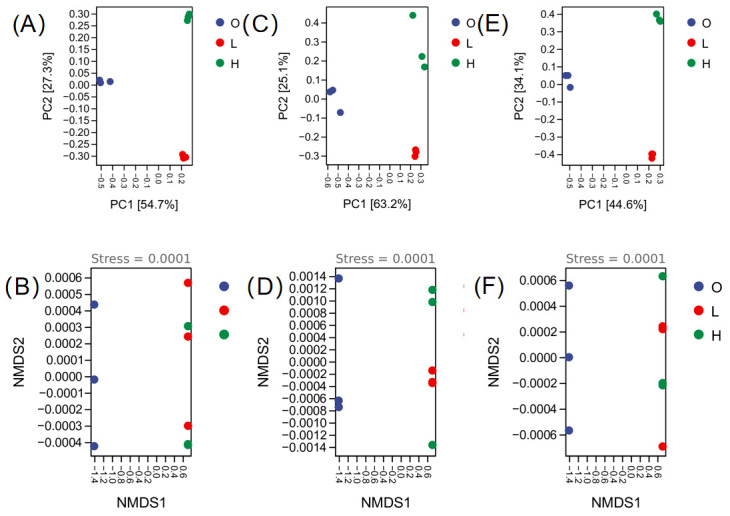
Beta diversity analysis of bacterial (16S), microeukaryotic (18S), and fungal (ITS) communities based on PCoA and NMDS ordinations. (**A**,**B**) 16S rRNA gene amplicon sequencing; (**C**,**D**) 18S rRNA gene amplicon sequencing; (**E**,**F**) ITS amplicon sequencing. (**A**,**C**,**E**) Principal coordinate analysis (PCoA) based on Bray–Curtis distance matrices. Percentages on the axes indicate the proportion of variance explained by each principal coordinate. (**B**,**D**,**F**) Non-metric multidimensional scaling (NMDS) based on Bray–Curtis distance matrices. Stress values are shown in each panel, with stress < 0.1 generally considered a good fit. For both PCoA and NMDS, ellipses represent 95% confidence intervals for each group. Samples are colored by group: blue, pre-culture baseline (Group O); green, low-nitrogen ponds (Group L); orange, high-nitrogen ponds (Group H). Significant differences in community structure between groups were assessed using permutational multivariate analysis of variance (PERMANOVA) based on Bray–Curtis distances (999 permutations), with *p* < 0.05 considered statistically significant.

Beta diversity was evaluated using PCoA and NMDS on Bray–Curtis matrices, with 95% confidence ellipses highlighting group dispersion (Figure 1). Variance decomposition showed marker-specific patterns: 16S amplicons had 82.0% total variance explained by PCoA1 (54.7%) and PCoA2 (27.3%); 18S showed stronger primary separation (PCoA1: 63.2%); ITS exhibited more balanced axes (PCoA1: 44.6%, PCoA2: 34.1%). These differences suggest variable sensitivity of markers to community structural gradients. In all three datasets, samples from the pre-culture baseline (Group O) were clearly separated from the low-nitrogen (Group L) and high-nitrogen (Group H) pond samples along the first principal coordinate, while Groups L and H clustered more closely together (Figure 1A,C,E). The 95% confidence ellipses further supported the distinct separation of Group O from the culture pond groups.

NMDS ordination showed a similar pattern, with Group O distinctly separated from Groups L and H along NMDS1 (Figure 1B,D,F). The stress values for all NMDS analyses were 0.0001 (well below the commonly accepted threshold of 0.1), indicating excellent representation of the original distances in two dimensions. The 95% confidence ellipses in the NMDS plots were consistent with those observed in the PCoA ordinations. Given the small sample size, NMDS stress values should be interpreted with caution; however, the observed pattern was consistent with the PCoA ordination and was further supported by the PERMANOVA results.

Inter-group differences in microbial community composition were detected across all three amplicon datasets (16S, 18S, ITS) using permutation-based multivariate analysis of variance (PERMANOVA, 999 permutations) with Bray–Curtis dissimilarity as the distance metric (16S: *R*^2^ = 0.342, *p* = 0.001; 18S: *R*^2^ = 0.287, *p* = 0.001; ITS: *R*^2^ = 0.315, *p* = 0.001). Although each group in this study contained only three biological replicates (which limits the generalizability of statistical inference), the degree of separation between groups observed in the PCoA and NMDS plots strongly suggests that aquaculture environments with different nitrogen levels shaped distinct microbial community structures.

### 3.4. Microbial Community Structure Analysis

**Figure 2:**
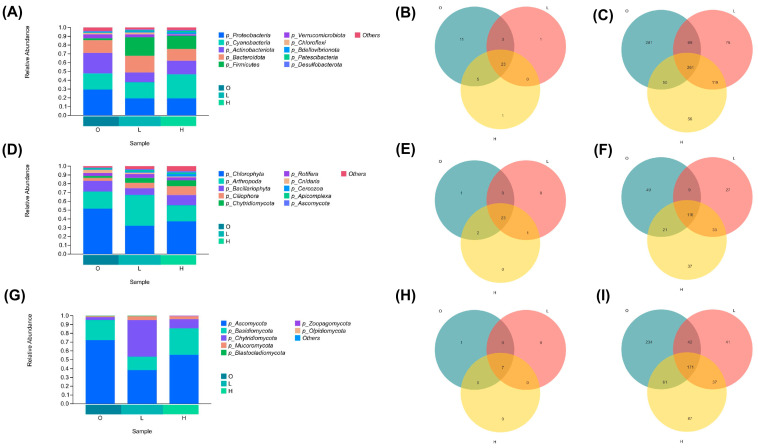
Taxonomic composition of bacterial (16S), microeukaryotic (18S), and fungal (ITS) communities. (**A**–**C**) 16S: (**A**,**B**) top 10 phyla; (**C**) top 10 genera. (**D**–**F**) 18S: (**D**,**E**) top 10 phyla; (**F**) top 10 genera. (**G**–**I**) ITS: (**G**,**H**) top 10 phyla; (**I**) top 10 genera. Groups: O, pre-culture baseline; L, low-nitrogen ponds; H, high-nitrogen ponds. Relative abundances are shown as proportions (%). Taxa < 1% are summarized as “Others”.

Bacterial communities (16S rRNA gene): At the phylum level, a total of 44 bacterial phyla were identified, with Proteobacteria (22.74%), Cyanobacteria (21.40%), Actinobacteriota (16.61%), Bacteroidota (15.48%), and Firmicutes (12.85%) being the dominant phyla (relative abundance > 10%) (Figure 2A). Intergroup comparisons revealed that the relative abundances of Proteobacteria and Actinobacteriota in the post-culture ponds (Groups L and H) decreased significantly compared with the pre-culture baseline (Group O) (Proteobacteria: from 29.58% to 19.18%; Actinobacteriota: from 23.44% to 11.30%). Conversely, Cyanobacteria were significantly enriched in the high-nitrogen group (Group H) (from 18.13% to 27.75%). Microbial diversity assessment at the genus rank revealed 911 distinct bacterial taxa, with 261 genera (28.6%) constituting the shared core microbiome across all three experimental groups (Figure 2C). The total number of OTUs was highest in the low-nitrogen group (Group L, 4108) and lowest in the high-nitrogen group (Group H, 3209) (Table 2), suggesting that moderate nitrogen levels may sustain higher bacterial species richness.

Microeukaryotic communities (18S rRNA gene): At the phylum level, a total of 27 microeukaryotic phyla were identified, with Chlorophyta (40.42%), Arthropoda (24.27%), Bacillariophyta (10.60%), and Ciliophora (6.35%) being the dominant phyla (Figure 2D). Intergroup comparisons showed that the relative abundance of Chlorophyta decreased significantly in the post-culture ponds (from 51.50% to 32.43%), whereas Arthropoda increased significantly in the low-nitrogen group (Group L) (from 19.79% to 34.98%). A total of 292 distinct microeukaryotic genera were detected at the genus rank, with 116 genera (39.7%) forming the conserved core assemblage shared across all three experimental conditions (Figure 2F). The total number of OTUs was lowest in the low-nitrogen group (Group L, 1428) and highest in the high-nitrogen group (Group H, 1535) (Table 2), a trend opposite to that observed in the bacterial communities.

Fungal communities (ITS region): Taxonomic classification at the phylum rank revealed eight distinct fungal lineages, with Ascomycota (55.36%), Basidiomycota (22.57%), and Chytridiomycota (18.45%) constituting the predominant phyla in the analyzed communities (Figure 2G). Intergroup comparisons indicated that the relative abundance of Ascomycota decreased significantly in the post-culture ponds (from 72.05% to 38.55–55.48%), while Chytridiomycota increased substantially after cultivation (from 3.06% to 41.66–10.63%), peaking in the low-nitrogen group (Group L). Basidiomycota decreased significantly in the low-nitrogen group (from 23.13% to 14.75%). At the genus level, a total of 673 fungal genera were identified, of which 171 were shared among the three groups (Figure 2I). The total number of OTUs was highest in the pre-culture baseline (Group O, 3163) and lowest in the low-nitrogen group (Group L, 2094) (Table 2), suggesting that aquaculture activities may reduce fungal species richness.

### 3.5. Microbial Community Annotation and Differential Abundance Analysis

**Figure 3:**
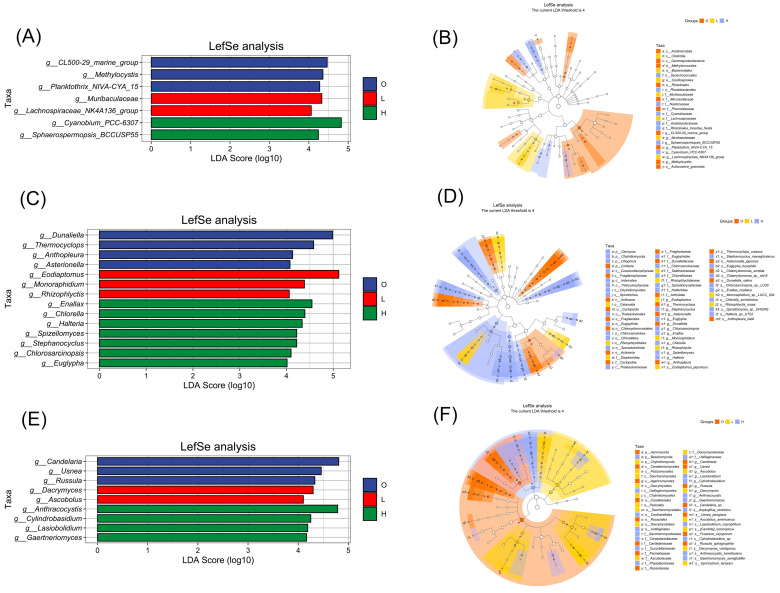
LEfSe analysis of differentially abundant microbial taxa. (**A**,**B**) 16S (bacteria); (**C**,**D**) 18S (microeukaryotes); (**E**,**F**) ITS (fungi). (**A**,**C**,**E**) LDA score histograms showing taxa with LDA scores > 4.0 (*p* < 0.05) among groups O (pre-culture baseline), L (low-nitrogen), and H (high-nitrogen). (**B**,**D**,**F**) Cladograms representing the taxonomic distribution of differentially abundant taxa. Circles from the center to the periphery represent taxonomic levels from phylum to genus. Colors indicate enrichment: red (H group), green (L group), blue (O group); yellow circles indicate non-significant taxa.

Linear Discriminant Analysis Effect Size (LEfSe) was used to identify statistically significant microbial biomarkers (represented by LDA scores) between groups and to compare relative abundances at different taxonomic levels (Figure 3B,D,F). It is worth noting that LEfSe identified candidate biomarkers, but these results need to be validated with a larger sample size. The numbers of biomarkers identified for the three groups were 187 (16S), 213 (18S), and 178 (ITS), respectively. Linear discriminant analysis (LDA) effect size measurement was employed to assess the magnitude of differential abundance for each taxon identified as significantly distinct among groups. In this study, LDA score ranges were: 2.8498 (L) to 4.8598 (L) for bacteria, 2.8012 (O) to 5.1285 (L) for microeukaryotes, and 2.7375 (L) to 5.2877 (L) for fungi, indicating both commonality and specificity in taxon abundance among different ponds. For a more in-depth comparison of the microbial composition among the three ponds, data with an LDA (Linear Discriminant Analysis) score threshold (log10) ≥ 4 in the LEfSe bar plots were chosen (Figure 3A,C,E). The analysis results demonstrated that the low-nitrogen pond was characterized by an enrichment of specific microbial taxa. Among bacteria, genera such as Muribaculaceae and *Lachnospiraceae_*NK4A136_group were abundant. In the eukaryotic domain, genera including *Eodiaptomus*, *Monoraphidium*, and *Rhizophlyctis* were prevalent. Additionally, in the fungal kingdom, genera like *Dacrymyces* and *Ascobolus* showed enrichment (Figure 3A). The high-nitrogen pond was enriched with bacterial genera such as *Cyanobium_*PCC-6307 and *Sphaerospermopsis_*BCCUSP55; microeukaryotic genera such as *Enallax*, *Chlorella*, *Halteria*, *Stephanocyclus*, *Spizellomyces*, *Chlorosarcinopsis*, and *Euglypha*, *Cylindrotheca*; and fungal genera such as *Anthracocystis*, *Cylindrobasidium*, *Lasiobolidium*, and *Gaertneriomyces* (Figure 3C). The control pond was enriched with bacterial taxa such as CL500-29_marine_group, *Methylocystis*, and *Planktothrix_*NIVA-CYA_15; eukaryotic genera such as *Dunaliella*, *Thermocyclops*, *Anthopleura*, and *Asterionella*; and fungal genera such as *Candelaria*, *Usnea*, and *Russula* (Figure 3E). These results suggest the presence of potential microbial biomarkers in bacterial, microeukaryotic, and fungal communities of *M. nipponense* aquaculture water under different nitrogen levels, with the enriched taxa in each group showing a distinct functional differentiation.

### 3.6. Functional Prediction of Microbial Communities

#### 3.6.1. FAPROTAX Functional Prediction

The Functional Annotation of Prokaryotic Taxa (FAPROTAX) algorithm was implemented to predict microbial functional capacities, encompassing a total of 64 ecological guilds across key biogeochemical processes. The overall functional profile was dominated by heterotrophic metabolism-related functions, accounting for 62.3–78.5% of the total functional abundance, followed by photoautotrophy (23.9–67.2%) and nitrogen cycling-related functions (1.2–3.5%). The core functions of the microbial community were organic matter decomposition and primary production, alongside participation in key biogeochemical cycles of elements. Ranked by average abundance across all samples, the high-abundance functional categories (average abundance > 1000) included: aerobic_chemoheterotrophy (5236.7 ± 892.4), chemoheterotrophy (5235.1 ± 891.8), oxygenic_photoautotrophy (3872.3 ± 610.5), photoautotrophy (3878.9 ± 611.2), and photosynthetic_cyanobacteria (3869.5 ± 609.8). Other functions with relatively high abundance were ureolysis (827.4 ± 215.6), methylotrophy (726.3 ± 189.2), and nitrate_reduction (689.5 ± 156.4), all important functions involved in nitrogen cycling or degradation of organic small molecules. One-way ANOVA with appropriate post hoc tests identified 22 functional categories showing significant differences among groups (Table 3). These significantly different functions, sorted by ascending ANOVA *p* value, were: methanotrophy, methylotrophy, nitrogen_fixation, photoheterotrophy, ureolysis, oxygenic_photoautotrophy, photoautotrophy, photosynthetic_cyanobacteria, phototrophy, hydrocarbon_degradation, aerobic_chemoheterotrophy, chlorate_reducers, dark_oxidation_of_sulfur_compounds, dark_sulfide_oxidation, chemoheterotrophy, methanol_oxidation, nitrate_respiration, nitrogen_respiration, fermentation, aerobic_ammonia_oxidation, nitrification, and nonphotosynthetic_cyanobacteria.

Among these, the nitrogen-cycling-related functions ureolysis and nitrification showed changes in the post-culture ponds. The predicted abundance of ureolysis was highest in the high-nitrogen group (Group H), consistent with the accumulation of ammonia nitrogen in the water. The predicted abundance of nitrification was higher in both Group H and the pre-culture baseline (Group O) than in the low-nitrogen group (Group L), partially aligning with the elevated ammonia and nitrite nitrogen levels observed in the high-nitrogen ponds. In contrast, the predicted abundances of nitrate_reduction and nitrite_respiration did not differ significantly among groups.

#### 3.6.2. Bacterial Phenotype Prediction

**Figure 4:**
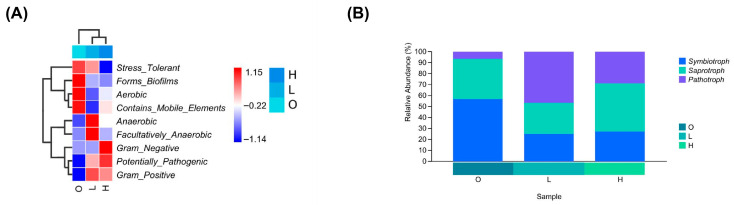
Predicted bacterial phenotypes and fungal functional guilds. (**A**) Heatmap of predicted bacterial phenotypes. Standardized functional abundance is shown by color (red: high, blue: low). Samples and functional traits were clustered based on similarity (Euclidean distance, UPGMA). Groups: O (pre-culture baseline), L (low-nitrogen), H (high-nitrogen). (**B**) Relative abundance (%) of fungal functional guilds predicted by FUNGuild. Significant differences among groups were determined by one-way ANOVA with Tukey’s HSD or Games–Howell post hoc tests (*p* < 0.05), indicated by different superscript letters.

Based on OTUs and alignment files, the relative abundances of nine bacterial phenotypes (Gram-positive, Gram-negative, Aerobic, Anaerobic, Facultatively Anaerobic, Biofilm Forming, Potentially Pathogenic, Mobile Elements, and Oxidative Stress Tolerant) were analyzed across the three groups (O, L, H) (Figure 4A). The statistical test results are presented in Supplementary Table S2.

In the pre-culture baseline group (Group O), phenotypes such as stress-tolerant, biofilm-forming, aerobic, and containing mobile elements showed relatively high abundances. Phenotypic enrichment patterns differed significantly among nitrogen treatments: Biofilm-forming and stress-tolerant phenotypes showed higher abundance in the medium-nitrogen group compared to both low-nitrogen (Group L) and high-nitrogen (Group H) treatments (*p* < 0.05). In Group L, facultative anaerobes were enriched relative to Group H (*p* < 0.05), while obligate anaerobes increased compared to the control (Group O; *p* < 0.05). Notably, potentially pathogenic phenotypes were more prevalent in Group H than in Group O (*p* < 0.05), with no significant difference observed between Groups H and L for this category. Furthermore, no nominally significant differences among the three groups were found for Gram-negative, aerobic, mobile element-containing, or Gram-positive phenotypes (*p* > 0.05). It should be noted that, due to only three biological replicates per group, the *p*-values from the above statistical tests should be considered exploratory results and should not be used as the basis for definitive inference. The statistical test results of BugBase bacterial phenotype prediction for water microbes in *Macrobrachium nipponense* culture ponds based on 16S rRNA gene data are presented in Supplementary Table S2.

#### 3.6.3. FUNGuild Functional Prediction

Taxonomic annotation of ITS-region ASVs was performed using the UNITE database to obtain composition profiles at various taxonomic levels. Metabolic functions of the fungal community were predicted using PICRUSt2, with functional pathway information retrieved from the KEGG and MetaCyc databases. On this basis, ecological functional guilds of the fungal community were assigned using FUNGuild, yielding 5020 ASVs with functional annotations, which were further screened to obtain 17 functional guilds (Table 4).

At the trophic mode level, the relative abundance of Symbiotroph was highest in the control group (56.58%) and lowest in the low-nitrogen group (25.25%), while the relative abundance of Saprotroph was highest in the high-nitrogen group (44.01%) and lowest in the low-nitrogen group (28.18%) (Figure 4B). The relative abundance of Pathotroph followed the order: low-nitrogen group (46.56%) > high-nitrogen group (28.73%) > control group (6.77%). These results suggest a higher potential risk of fungal pathogens in the low-nitrogen group.

At the guild level, Plant Pathogen exhibited the highest relative abundance, accounting for 48.14% and 28.66% in the low-nitrogen and high-nitrogen groups, respectively. ANOVA showed a nominal *p*-value of less than 0.01 for the difference between groups. However, given the small sample size of this study (only three biological replicates per group), this difference should be regarded as a preliminary, exploratory observation that requires validation in studies with larger sample sizes. Further analysis of the enrichment of Animal Pathogen and Fungal Parasite across different ponds revealed that the highest abundance of Animal Pathogen occurred in the low-nitrogen group (0.73% ± 1.02%), while the highest abundance of Fungal Parasite occurred in the high-nitrogen group (0.22% ± 0.22%); Under the current small-sample condition, the differences between groups did not reach the nominal level of statistical significance (*p* > 0.05).

### 3.7. Correlation Analysis with Environmental Factors

Redundancy analysis (RDA) was applied to evaluate the influence of four nitrogen-related environmental variables on the compositional patterns of aquatic microbial communities. RDA was first conducted at the phylum level; permutation tests yielded (*p* > 0.05), indicating no significant correlation between phylum-level community structure and the measured nitrogen factors, suggesting that the phylum level may not be sufficiently refined to reveal detailed microbial–environment linkages.

**Figure 5:**
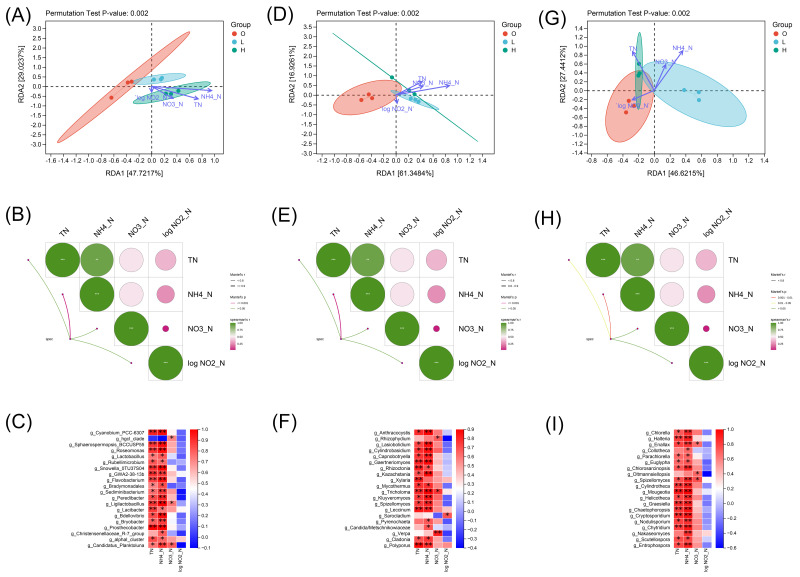
Associations between microbial communities and environmental factors. (**A**–**C**) 16S rRNA gene-based analyses: (**A**) RDA ordination plot showing relationships between bacterial communities and environmental factors. Environmental factors (TN, NH_4_^+^-N, NO_3_^−^-N) were Z-score standardized; NO_2_^−^-N was log-transformed due to low variance. Species data were Hellinger-transformed. Vectors indicate environmental factors; points are colored by group (O: red, L: blue, H: green). (**B**) Mantel test heatmap showing correlations between bacterial community distance (Bray–Curtis) and environmental distance (Euclidean, with NO_2_^−^-N log-transformed). Color intensity indicates correlation strength. (**C**) Spearman’s rank correlation heatmap showing relationships between dominant bacterial genera and environmental factors. Color intensity indicates correlation strength; asterisks indicate significance (* *p* < 0.05, ** *p* < 0.01). (**D**–**F**) 18S rRNA gene-based analyses: (**D**) RDA ordination plot showing relationships between eukaryotic communities and environmental factors. Environmental factors (TN, NH_4_^+^-N, NO_3_^−^-N) were Z-score standardized; NO_2_^−^-N was log-transformed due to low variance. Species data were Hellinger-transformed. Vectors indicate environmental factors; points are colored by group (O: red, L: blue, H: green). (**E**) Mantel test heatmap showing correlations between eukaryotic community distance (Bray–Curtis) and environmental distance (Euclidean, with NO_2_^−^-N log-transformed). Color intensity indicates correlation strength. (**F**) Spearman’s rank correlation heatmap showing relationships between dominant eukaryotic genera and environmental factors. Color intensity indicates correlation strength; asterisks indicate significance (* *p* < 0.05, ** *p* < 0.01, *** *p* < 0.001). (**G**–**I**) ITS gene-based analyses: (**G**) RDA ordination plot showing relationships between fungal communities and environmental factors. Environmental factors (TN, NH_4_^+^-N, NO_3_^−^-N) were Z-score standardized; NO_2_^−^-N was log-transformed due to low variance. Species data were Hellinger-transformed. Vectors indicate environmental factors; points are colored by group (O: red, L: blue, H: green). (**H**) Mantel test heatmap showing correlations between fungal community distance (Bray–Curtis) and environmental distance (Euclidean, with NO_2_^−^-N log-transformed). Color intensity indicates correlation strength. (**I**) Spearman’s rank correlation heatmap showing relationships between dominant fungal genera and environmental factors. Color intensity indicates correlation strength; asterisks indicate significance (* *p* < 0.05, ** *p* < 0.01). Significance in all tests was assessed using 999 permutations.

To elucidate finer-scale ecological patterns, subsequent analyses examined the correspondence between microbial community structure and environmental variables at the genus level. The total explained variation in species distribution by the environmental factors was 76.75% for bacteria (16S), 78.27% for microeukaryotes (18S), and 74.06% for fungi (ITS) (Figure 5A,D,G). Permutation test results (Pr) showed that only NH_4_-N had a significant effect on the community distribution of bacteria (Pr = 0.006), fungi (Pr = 0.009), and microeukaryotes (Pr = 0.006), whereas total nitrogen (TN), nitrate nitrogen (NO_3_-N), and nitrite nitrogen log NO_2_-N showed no significant correlation with any of the three microbial groups, indicating that NH_4_-N was the core nitrogen factor driving microbial community differentiation.

Mantel test results revealed that the bacterial community (16S) exhibited the strongest correlation with environmental factors, while the Mantel correlation coefficients for microeukaryotes (18S) and fungi (ITS) were relatively lower (Figure 5B,E,H), suggesting differential responsiveness among microbial groups to environmental factors.

In the genus-level correlation heatmaps: for bacteria, dominant genera such as *Cyanobium_*PCC-6307, *Sphaerospermopsis_*BCCUSP55, *Shinella*, and *Bradyrhizobium* showed significant positive correlations with TN, NH_4_-N, and NO_3_-N, whereas only *Candidatus Saccharimonas* exhibited a weak negative correlation with environmental factors (Figure 5C). For microeukaryotes, dominant taxa including *Chlorella*, *Emiliania*, *Phaeocystis*, and *Chlorodendrophyceae* were generally significantly positively correlated with TN, NH_4_-N, and NO_3_-N (Figure 5F). For fungi, the majority of dominant genera such as Anthracocystis, Lunulospora, and Candida were significantly positively correlated with environmental factors, while a few taxa, such as Candidatus Pseudovacuolata and Cylindrocarpon, exhibited weak negative correlations (Figure 5I).

Notably, although NH_4_-N was identified as a significant driving factor, its independent contribution to community variation remained limited. This suggests that unmeasured environmental factors—such as dissolved oxygen or organic matter content—or indirect effects of NH_4_-N (e.g., influencing pH or the availability of other nutrients) may collectively shape the structure of microbial communities.

## 4. Discussion

The microbial community in aquaculture water is a core functional unit of the ecosystem, playing an irreplaceable role in material cycling, energy flow, and maintaining environmental homeostasis [24]. The dominant microorganisms identified in this study were similar to those reported in ponds for other aquatic animals. Within the bacterial domain, the predominant microbial taxa frequently encountered in freshwater aquaculture ponds include Proteobacteria, Actinobacteriota, and Bacteroidota [25,26]. Cyanobacteria also occupy an important position in freshwater ponds, for instance, maintaining dominance in shrimp polyculture ponds [27] and in grass carp (*Ctenopharyngodon idella*) ponds during later cultivation stages [28]. Regarding microeukaryotes, the typical dominant groups vary considerably among different aquaculture ponds, with Chlorophyta, Ciliophora, Bacillariophyta, and Arthropoda being common dominant groups [25,29,30]. In the realm of fungal research, Ascomycota, Basidiomycota, and Chytridiomycota frequently emerge as the principal dominant phyla within aquaculture pond ecosystems [31,32].

Microorganisms can degrade organic wastes like residual feed and feces, participate in the transformation of toxic substances such as ammonia and nitrite, thereby reducing water pollution risks [33,34]. The microbial diversity and community architecture in aquatic systems are of fundamental importance for the preservation of ecological functionalities. Excessively low microbial diversity often indicates environmental instability [35], while changes in the abundance of functional groups (e.g., nitrifying bacteria, photosynthetic algae) are linked to the nutritional supply and health protection of cultured organisms [36]. Proteobacteria are core groups in nitrogen cycling and organic matter degradation [14,37]; Bacteroidota possess high efficiency in decomplexing polysaccharides, proteins, and other complex organic materials [38]; Actinobacteriota exhibit a high proficiency in breaking down refractory organic compounds and are actively involved in the processes of nitrogen and phosphorus mineralization [39]. Together, these three phyla constitute the core system for organic matter decomposition and nutrient transformation in ponds. Cyanobacteria are major contributors to primary productivity [40], capable of photoautotrophic organic synthesis. However, sustained high abundance of cyanobacteria may lead to cyanobacterial blooms, thereby harming cultured organisms [41]. Ascomycota and Basidiomycota are primarily saprophytic, participating in the decomposition of recalcitrant organic matter like lignin and cellulose in pond sediment and water, which positively impacts substrate improvement [42,43]. The degradative activity of Chytridiomycota on algal detritus can, to some extent, alleviate eutrophication pressure [44], although the presence of some pathogenic chytrids may pose a potential threat to cultured animals [45]. Furthermore, Chlorophyta and Bacillariophyta help maintain dissolved oxygen balance through photosynthesis, while Arthropoda, as primary consumers, reflect phytoplankton abundance through their population dynamics. The collaborative interactions among these microorganisms facilitate the sustained stability of the pond’s food web dynamics.

In this study, the composition of dominant bacterial phyla remained consistent, but the relative abundances of some taxa showed significant differences, suggesting that aquaculture activities and changes in nitrogen nutrition may have promoted the succession of microbial communities. At the bacterial level, the significant increase in the relative abundance of Cyanobacteria after cultivation may result from the combined effects of external environmental conditions and internal pond nutrient status. On one hand, nutrient inputs (N, P, etc.) from farming activities may create eutrophic conditions favorable for cyanobacterial growth [46,47]. On the other hand, seasonal changes (e.g., light, water temperature) might also be important driving factors [37]. The decline in Proteobacteria and Actinobacteriota may reflect shifts in organic matter degradation pathways or competitive dynamics as cultivation progresses. For example, with the accumulation of residual feed and feces, the available complex organic matter decreases, leading to a decline in the abundance of taxa that depend on it [48,49]. Among microeukaryotes, the reduction in Chlorophyta might be related to resource competition (e.g., with Cyanobacteria) or grazing pressure from zooplankton [50,51]. Additionally, the increase in Arthropoda (mainly zooplankton groups) in the low-nitrogen pond might suggest that the low-nitrogen environment altered the composition of primary producers, providing a more suitable food source for them [50,52]. The fungal community structure exhibited a more marked response. Specifically, the relative abundances of Ascomycota and Basidiomycota demonstrated a declining pattern within the low-nitrogen treatment group, potentially suggesting that nitrogen buildup influenced their metabolic processes or competitive abilities [53]. In contrast, Chytridiomycota displayed a significant surge in relative abundance following cultivation, marking it as one of the most prominent alterations observed. Molts and carcasses of crustaceans during cultivation may provide unique substrates for chytrids [44], giving them a competitive advantage for resources, while parasitism of algae by some chytrid groups might also promote this process [54,55]. It should be noted that the pre-culture baseline water samples were collected from an external river rather than from the same ponds. Inherent differences in hydrology, nutrient backgrounds, and microbial composition exist between river and pond systems. Therefore, the conclusions regarding temporal succession should be regarded as exploratory observations rather than definitive cultivation effects. Nevertheless, the comparison between the low-nitrogen and high-nitrogen groups after the culture period (both with consistent sampling backgrounds) can still reliably reflect the effects of different nitrogen levels on the microbial community.

Based on the key differential species identified by LEfSe analysis, this study revealed indicator species with ecological significance across three levels: bacteria, microeukaryotes, and fungi. At the bacterial level, the cyanobacterial genus *Cyanobium_*PCC-6307 showed an enrichment trend in the low-nitrogen pond. *Cyanobium* is one of the most widespread picocyanobacteria, found in freshwater, brackish, and marine environments [56]. Studies report that *Cyanobium* can deplete environmental nitrogen to very low levels [57] and efficiently capture faint and spectrally differentiated underwater light by adjusting the composition of accessory pigments like phycoerythrin and phycocyanin [58,59], granting it a competitive advantage. Additionally, some *Cyanobium* strains possess non-heterocystous nitrogen fixation capabilities, allowing them to overcome nitrogen limitation by fixing atmospheric N_2_ [57]. In this study, *Cyanobium_*PCC-6307 was a biomarker distinguishing the low-nitrogen pond from the other two groups, likely possessing similar functions that confer an advantage in low-nutrient conditions. At the eukaryotic level, the genus *Eodiaptomus* showed an enrichment trend in the high-nitrogen pond. Research indicates that *Eodiaptomus* population fluctuations may be driven by food availability and predation [60], and its population dynamics are sensitive to environmental changes, declining significantly under eutrophic conditions [61]. In this study, the significant enrichment in the high-nitrogen pond suggests that abundant plankton provided ample food resources, enabling population expansion [61]. However, its identification as a biomarker for the high-nitrogen pond may indicate that nutrient levels in our high-nitrogen ponds were far below eutrophic thresholds. At the fungal level, the genus *Anthracocystis* showed an enrichment trend in the high-nitrogen cultivation pond. *Anthracocystis* has been previously documented as a strict parasite of terrestrial grasses (Poaceae), obtaining nutrients by infecting hosts and distributed only terrestrially [62,63]. This study is the first to detect enrichment of this fungal genus in the water environment of a high-nitrogen aquaculture pond. It is speculated that it may have adapted to the high-nitrogen, high-organic conditions of the pond through metabolic plasticity [64,65,66], or it relies on unidentified hosts or associated organisms in the aquatic environment for proliferation [55,67]. The specific mechanisms require further verification.

In aquaculture systems, the microbial-driven nitrogen cycling process is a key link in maintaining water ecological functions. In this study, FAPROTAX was used to predict microbial functions related to nitrogen transformation. It was found that the predicted abundance of ureolysis in the high-nitrogen treatment group tended to be higher than that in the low-nitrogen group and the pre-culture baseline group (Table 3), and this group also showed significant accumulation of ammonia nitrogen (NH_4_^+^-N). The increase in predicted ureolysis abundance may be associated with higher organic matter content (e.g., residual feed and feces) in the high-nitrogen group, which provides ample substrates for ureolytic bacteria. As ureolysis is a key process in the conversion of organic nitrogen to ammonia nitrogen [68], its enhanced activity leads to ammonia accumulation in the high-nitrogen group. Meanwhile, the predicted abundance of nitrification did not increase synchronously in the high-nitrogen group, possibly indicating inhibition of nitrification function in aquaculture water with high ammonia concentrations. Accumulating evidence indicates that nitrification processes are modulated by a complex interplay of environmental factors. Elevated ammonia-nitrogen levels induce the formation of free ammonia (FA), which exerts direct cytotoxic effects on the metabolic functions of both ammonia-oxidizing bacteria (AOB) and ammonia-oxidizing archaea (AOA). This biochemical inhibition subsequently disrupts the initiation and propagation of nitrification pathways, as evidenced by previous mechanistic studies [69,70]. In addition, key environmental factors such as dissolved oxygen and pH in the water may not meet the optimal requirements for normal nitrifier metabolism, indirectly limiting nitrification function and blocking ammonia oxidation [71]. Furthermore, no significant differences in nitrate reduction or nitrite respiration were observed among groups, suggesting that denitrification processes may be more strongly regulated by factors such as organic carbon content and redox potential, rather than directly responding to the ammonia gradient [72]. The accumulation of nitrite nitrogen in the high-nitrogen group, together with the slightly higher mean values of denitrification-related functions in this group, further indicates possible incomplete denitrification or insufficient nitrite reduction capacity, leading to the accumulation of nitrite nitrogen due to inefficient conversion to inert gases such as dinitrogen [73]. It should be noted that the above analyses are based on functional predictions from FAPROTAX, which reflect the potential metabolic capacity of the microbial community rather than actual metabolic rates. FAPROTAX works by matching taxonomic units obtained from 16S rRNA gene sequencing to a manually curated reference database, thereby predicting the functional traits that the sample may possess. Its accuracy is limited by the coverage of the reference database and the resolution of taxonomic annotation [74]. Therefore, the interpretations of nitrogen-cycling functions in this study should be considered hypothetical.

Prior research has revealed notable and statistically significant positive associations between microbial community composition and the concentrations of total nitrogen, ammonium nitrogen, nitrate, and nitrite in the environment [75,76,77]. Consistent with prior research, ammonium nitrogen (NH_4_-N) was the core factor regulating the microbial community in this study [78]. However, no significant correlation was found between other nitrogen forms and microorganisms, possibly because ammonium nitrogen was the predominant form in this study, and fluctuations in other forms were low, with no significant differences among groups. The strongest correlation between bacteria and nitrogen factors in this study may stem from bacteria’s ability to directly utilize ammonium nitrogen (NH_4_-N) [79], making their response to nitrogen gradient changes more direct and sensitive. Fungi are mostly saprophytic, and their nutrient acquisition relies more on organic matter decomposition [33]; nitrogen is often supplied indirectly via organic matter, resulting in a weaker direct correlation with inorganic nitrogen gradients. Microeukaryotes are simultaneously constrained by non-nitrogen factors such as light, carbon sources, and predation pressure [60,80]. These factors may weaken their direct association with nitrogen gradients, leading to a less significant response compared to bacteria. It is important to note that, based only on correlation analyses (RDA, Mantel tests), this study cannot establish causality between ammonia nitrogen and microbial community shifts, but merely suggests a strong association.

Furthermore, no significant correlations between microbial communities and nitrogen factors (e.g., ammonia nitrogen) were detected at the phylum level in this study, suggesting that the response of microbial communities to nitrogen nutrition may occur more at the genus level or lower taxonomic levels rather than at the phylum level. This issue requires more refined analyses in future studies to clarify. This study focused exclusively on nitrogen-related parameters (total nitrogen, ammonia nitrogen, nitrate, and nitrite), while other important environmental variables that may influence microbial community structure—such as water temperature, dissolved oxygen, pH, total phosphorus, and organic carbon content—were not measured. These variables have been shown in previous studies to significantly regulate microbial communities [75,81]. Consequently, the observed associations between nitrogen forms and microbial communities might be partially confounded or modulated by these unmeasured variables. For example, fluctuations in dissolved oxygen may affect the consistency between the predicted abundance of nitrification functions and actual activity [82]. Another key limitation of this study is the small sample size (only three biological replicates per group). Although this is common in pilot or exploratory microbiome studies, it limits the statistical power of beta diversity comparisons, biomarker discovery (LEfSe), and functional prediction analyses. Small group sample sizes are particularly susceptible to the influence of outliers and may not represent true population-level variation. Given the exploratory nature of the current dataset, these findings should be interpreted as initial evidence requiring further validation. Subsequent investigations employing expanded sample cohorts are essential to confirm the observed ecological patterns and enhance statistical robustness.

## 5. Conclusions

In this study, multi-omics sequencing technology was employed for the first time to compare the microbial community changes in *Macrobrachium nipponense* ponds between the pre-culture and culture periods, to characterize the water microbial community structure, to predict microbial functions, and to analyze the associations between microorganisms and water quality parameters. The results showed that the dominant microorganisms in the pond water during the culture period were Proteobacteria, Cyanobacteria, Ascomycota, Basidiomycota, Chlorophyta, and Arthropoda. Aquaculture activities reduced the relative abundances of Ascomycota, Basidiomycota, and Chlorophyta to some extent, while increasing the relative abundances of Cyanobacteria, Chytridiomycota, and zooplankton. Functional predictions indicated that heterotrophic metabolism-related functions dominated the functional profile. The abundance of fungal pathogens was significantly higher in the low-nitrogen group, while potentially pathogenic bacteria were enriched in the high-nitrogen group. Correlation analysis with physicochemical factors revealed that ammonia nitrogen was an important driver of the microbial community, with the sensitivity to water quality factors ranking as follows: bacteria > fungi > microeukaryotes. This study preliminarily reveals the structure and dynamics of the microbial community in *M. nipponense* culture ponds, which holds significant ecological implications and practical value for the intensive management of this species. Monitoring the dynamic changes in ammonia nitrogen concentration and key microbial indicator groups (e.g., *Cyanobium_*PCC-6307 in low-nitrogen ponds, *Eodiaptomus* and *Anthracocystis* in high-nitrogen ponds) can serve as effective biological indicators for assessing pond water ecological status and water quality risks. Future studies should extend the observation period, use pre-stocking water from the same ponds as the baseline control, simultaneously collect and analyze sediment and gut microbial communities, comprehensively monitor environmental factors such as temperature, pH, and phosphorus, and integrate multi-omics approaches including metagenomics and metatranscriptomics. These efforts will further elucidate the assembly mechanisms, ecological functions, and complex interactions of microbial communities with environmental factors in aquaculture settings, thereby providing a more solid scientific basis for the healthy and sustainable development of *M. nipponense* aquaculture. The findings offer new technical approaches for water quality regulation and ecological remediation in *M. nipponense* ponds, and provide a scientific theoretical basis for water quality management in the culture of other freshwater shrimp and crab species, thereby promoting the green, healthy, and sustainable development of the aquaculture industry.

## Figures and Tables

**Table 1:** Data on Physicochemical Factors of Different Ponds.

**SAMPLE**	**TN**	**NH_4_** **^+^**	**NO_3_^−^**	**NO_2_^−^**
1	1.07 ± 0.16 ^ab^	0.64 ± 0.01 ^a^	0.29 ± 0.05 ^ab^	0.02 ± 0.002 ^a^
2	1.02 ± 0.23 ^ab^	0.56 ± 0.01 ^b^	0.28 ± 0.03 ^ab^	0.02 ± 0.004 ^a^
3 (L)	0.97 ± 0.13 ^ab^	0.64 ± 0.01 ^a^	0.27 ± 0.03 ^ab^	0.02 ± 0.001 ^a^
4	1.25 ± 0.18 ^b^	0.64 ± 0.01 ^a^	0.34 ± 0.02 ^a^	0.02 ± 0.003 ^a^
5	1.02 ± 0.15 ^ab^	0.64 ± 0.02 ^a^	0.31 ± 0.07 ^ab^	0.03 ± 0.005 ^b^
6 (H)	1.77 ± 0.20 ^c^	0.78 ± 0.01 ^c^	0.31 ± 0.06 ^ab^	0.03 ± 0.006 ^b^
O	0.86 ± 0.10 ^a^	0.34 ± 0.05 ^abc^	0.22 ± 0.08 ^b^	0.03 ± 0.001 ^ab^

Note: Abbreviations: TN, total nitrogen; L, low-nitrogen pond; H, high-nitrogen pond; O, original (control) pond. Values are presented as mean ± standard deviation (*n* = 3 per pond type). Groups 1–6 represent six parallel culture ponds after 30 days of stocking; group O represents the pre-culture water samples collected from the external river. Based on total nitrogen concentrations, groups 3 and 6 were selected as the low-nitrogen (L) and high-nitrogen (H) groups, respectively, for subsequent microbial diversity analysis. Different superscript letters within the same column indicate significant differences among groups as determined by one-way analysis of variance (ANOVA) followed by Tukey’s HSD post hoc test (*p* < 0.05). Unit: mg/mL.

**Table 2:** Richness and diversity indexes relative to each sample.

**SAMPLE ID**		**O1**	**O2**	**O3**	**L1**	**L2**	**L3**	**H1**	**H2**	**H3**
COUNTS	16S	55,886	58,196	56,981	83,397	74,553	71,283	69,538	78,264	65,556
	18S	65,133	61,504	64,777	81,128	77,806	76,120	79,494	75,156	80,164
	ITS	70,917	70,171	69,320	72,857	73,773	63,124	77,674	72,314	67,843
ALPHA DIVERSITY OBSERVED OTUS	16S	1372	1469	1131	1436	1371	1301	1141	1117	951
	18S	536	418	491	482	483	463	590	485	460
	ITS	1144	957	1062	808	674	612	899	766	695
CHAO1	16S	1373.9	1474.66	1133.99	1502.19	1390	1321.45	1156.16	1149.1	958.619
	18S	537.75	418.111	491.84	483.833	485.758	464.2	593.077	486.964	464
	ITS	1148.61	960.759	1064.65	813.308	674.554	612.2	904.328	770.6	695.72
SHANNON	16S	8.27061	8.25704	7.81716	8.78169	8.83757	8.90646	8.13512	8.0395	7.89473
	18S	6.33695	5.79592	6.67664	5.75124	6.14152	5.77031	7.1855	6.43951	6.02456
	ITS	7.30058	6.79642	7.47145	7.37476	7.09398	6.80275	7.49179	7.20539	7.12332
SIMPSON	16S	0.991767	0.986814	0.987886	0.995036	0.99571	0.995569	0.991062	0.990356	0.989697
	18S	0.96065	0.948235	0.979153	0.900853	0.983498	0.921844	0.983595	0.956087	0.93147
	ITS	0.969749	0.96173	0.982396	0.983498	0.979428	0.974961	0.97849	0.972129	0.974081
GOODS COVERAGE	16S	0.999374	0.998943	0.999315	0.99726	0.998493	0.998591	0.998748	0.998258	0.999178
	18S	0.999856	0.999946	0.999874	0.999802	0.999749	0.999838	0.999713	0.999802	0.999713
	ITS	0.999528	0.999511	0.999595	0.999595	0.999848	0.999933	0.999561	0.999595	0.999848

Note: Alpha diversity indices were calculated based on rarefied ASV tables. Samples O1–O3: external river samples (pre-culture baseline); L1–L3: low-nitrogen pond group; H1–H3: high-nitrogen pond group. Counts indicates the number of sequences retained after removing singleton ASVs (i.e., the Non-singleton values from Supplementary Table S1). Observed OTUs represents the number of observed ASVs per sample. Chao1 estimates species richness, Shannon and Simpson indices reflect species diversity, and Good’s coverage indicates the sequencing completeness. Higher Good’s coverage values (close to 1) suggest that the sequencing depth is sufficient to capture the majority of microbial diversity in the samples.

**Table 3:** Comparison of significantly different functional abundances among three groups based on FAPROTAX annotation.

**RANK**	**FUNCTION**	**L**	**H**	**O**	**ANOVA** ***p***
1	methanotrophy	0.67 ± 1.15 ^a^	0.00 ± 0.00 ^a^	4082.67 ± 587.08 ^b^	<0.001
2	methylotrophy	755.33 ± 200.12 ^a^	408.67 ± 103.16 ^a^	5444.33 ± 809.04 ^b^	<0.001
3	nitrogen_fixation	70.00 ± 26.76 ^a^	20.67 ± 20.21 ^a^	2462.33 ± 556.12 ^b^	<0.001
4	photoheterotrophy	1045.00 ± 242.52 ^b^	278.00 ± 19.16 ^b^	25.67 ± 7.09 ^a^	<0.001
5	ureolysis	723.00 ± 100.21 ^b^	1624.00 ± 37.04 ^c^	236.00 ± 70.57 ^a^	<0.001
6	oxygenic_photoautotrophy	9804.33 ± 1410.00 ^a^	17,667.67 ± 1629.90 ^b^	5245.00 ± 2442.11 ^a^	<0.001
7	photoautotrophy	9814.67 ± 1410.11 ^a^	17,685.67 ± 1631.11 ^b^	5251.00 ± 2441.50 ^a^	<0.001
8	photosynthetic_cyanobacteria	9804.33 ± 1409.99 ^a^	17,667.67 ± 1629.90 ^b^	5245.00 ± 2442.11 ^a^	<0.001
9	phototrophy	10,849.33 ± 1591.11 ^b^	17,945.67 ± 1640.11 ^c^	5273.00 ± 2441.11 ^a^	<0.001
10	hydrocarbon_degradation	0.67 ± 1.15 ^a^	0.00 ± 0.00 ^a^	4087.00 ± 589.11 ^b^	<0.001
11	aerobic_chemoheterotrophy	5419.33 ± 1139.61 ^b^	6075.00 ± 337.12 ^b^	2350.00 ± 263.25 ^a^	0.001
12	chlorate_reducers	10.67 ± 11.02 ^a^	13.00 ± 19.92 ^a^	151.67 ± 47.00 ^b^	0.002
13	dark_oxidation_of_sulfur_compounds	10.33 ± 5.51 ^a^	4.00 ± 4.58 ^a^	42.33 ± 11.02 ^b^	0.002
14	dark_sulfide_oxidation	8.33 ± 4.93 ^ns^	0.00 ± 0.00 ^ns^	39.00 ± 14.11 ^ns^	0.003
15	chemoheterotrophy	14,352.00 ± 1019.38 ^ab^	11,690.00 ± 523.70 ^b^	9025.67 ± 1535.66 ^a^	0.003
16	methanol_oxidation	754.67 ± 199.89 ^a^	408.67 ± 103.16 ^a^	1361.67 ± 247.11 ^b^	0.003
17	nitrate_respiration	35.33 ± 19.16 ^ns^	57.33 ± 4.16 ^ns^	217.67 ± 60.11 ^ns^	0.003
18	nitrogen_respiration	35.33 ± 19.16 ^ns^	57.33 ± 4.16 ^ns^	217.67 ± 60.11 ^ns^	0.003
19	fermentation	8156.00 ± 2228.71 ^b^	5226.00 ± 253.35 ^b^	1111.67 ± 965.26 ^a^	0.003
20	aerobic_ammonia_oxidation	6.00 ± 5.29 ^a^	94.33 ± 35.33 ^b^	85.67 ± 17.90 ^b^	0.006
21	nitrification	6.00 ± 5.29 ^a^	94.33 ± 35.33 ^b^	85.67 ± 17.90 ^b^	0.006
22	nonphotosynthetic_cyanobacteria	88.33 ± 23.76 ^b^	50.00 ± 6.00 ^ab^	17.67 ± 17.33 ^a^	0.007

Note: Groups L, H, and O represent low-nitrogen ponds, high-nitrogen ponds, and the pre-culture baseline (external river), respectively. Functional abundances were predicted using FAPROTAX based on 16S rRNA gene amplicon sequencing data. Values are presented as mean ± standard deviation (*n* = 3 per group). One-way analysis of variance (ANOVA) was used for overall comparisons among the three groups. For functions with homogeneous variances (Levene’s test, *p* ≥ 0.05), Tukey’s HSD post hoc test was applied; for those with heterogeneous variances (*p* < 0.05), the Games–Howell post hoc test was applied. Different superscript letters (a, b, c) within the same row indicate statistically significant differences between groups (*p* < 0.05); “ns” indicates that although the overall ANOVA (*p* < 0.05), no pairwise comparison reached statistical significance. Only functions with ANOVA (*p* < 0.05) are listed.

**Table 4:** FUNGuild-Based Fungal Functional Prediction Results.

**RANK**	**GUILD**	**O**	**L**	**H**	**ANOVA** ***p***
1	Plant Pathogen	0.058258 ± 0.005432 ^a^	0.481438 ± 0.046903 ^c^	0.286557 ± 0.033967 ^b^	<0.01
2	Undefined Saprotroph	0.287462 ± 0.185328 ^ns^	0.156347 ± 0.017335 ^ns^	0.359425 ± 0.015565 ^ns^	0.141
3	Lichenized	0.406857 ± 0.124444 ^ab^	0.125466 ± 0.022634 ^a^	0.202134 ± 0.025005 ^b^	0.013
4	Ectomycorrhizal	0.163402 ± 0.044356 ^b^	0.132773 ± 0.026215 ^ab^	0.078809 ± 0.002051 ^a^	0.038
5	Wood Saprotroph	0.069658 ± 0.053947 ^ns^	0.088094 ± 0.009401 ^ns^	0.051956 ± 0.030214 ^ns^	0.535
6	Animal Pathogen	0.008027 ± 0.003670 ^ns^	0.007271 ± 0.010193 ^ns^	0.007043 ± 0.001713 ^ns^	0.984
7	Arbuscular Mycorrhizal	0.001053 ± 0.000947 ^ns^	0.007131 ± 0.004593 ^ns^	0.002494 ± 0.001018 ^ns^	0.109
8	Animal Endosymbiont	0.000056 ± 0.000097 ^ns^	0.000461 ± 0.000201 ^ns^	0.005533 ± 0.005174 ^ns^	0.151
9	Soil Saprotroph	0.001534 ± 0.001314 ^ns^	0.000578 ± 0.000115 ^ns^	0.002013 ± 0.000237 ^ns^	0.148
10	Leaf Saprotroph	0.001863 ± 0.000490 ^b^	0.000012 ± 0.000020 ^a^	0.001293 ± 0.000509 ^b^	0.005
11	Fungal Parasite	0.000701 ± 0.000727 ^ns^	0.000167 ± 0.000137 ^ns^	0.002183 ± 0.002217 ^ns^	0.253
12	Dung Saprotroph	0.000615 ± 0.000796 ^ns^	0.000000 ± 0.000000 ^ns^	0.000000 ± 0.000000 ^ns^	0.286
13	Endophyte	0.000326 ± 0.000054 ^b^	0.000037 ± 0.000029 ^a^	0.000243 ± 0.000328 ^ab^	0.351
14	Plant pathogenic on pollen	0.000083 ± 0.000063 ^ns^	0.000222 ± 0.000156 ^ns^	0.000043 ± 0.000038 ^ns^	0.158
15	Lichen Parasite	0.000000 ± 0.000000 ^a^	0.000000 ± 0.000000 ^a^	0.000222 ± 0.000106 ^b^	0.003
16	Plant Saprotroph	0.000078 ± 0.000134 ^ns^	0.000000 ± 0.000000 ^ns^	0.000037 ± 0.000059 ^ns^	0.572
17	Epiphyte	0.000028 ± 0.000048 ^ns^	0.000000 ± 0.000000 ^ns^	0.000012 ± 0.000021 ^ns^	0.565

Note: Groups O, L, and H represent the control group (external river water), low-nitrogen ponds, and high-nitrogen ponds, respectively. Functional guilds were assigned using FUNGuild based on ITS region amplicon sequencing data. Values are presented as mean ± standard deviation (*n* = 3 per group). One-way analysis of variance (ANOVA) was used for overall comparisons among the three groups. For functional guilds with homogeneous variances (Levene’s test, *p* ≥ 0.05), Tukey’s HSD post hoc test was applied; for those with heterogeneous variances (*p* < 0.05), the Games–Howell post hoc test was applied. Different superscript letters (a, b, c) within the same row indicate statistically significant differences between groups (*p* < 0.05); “ns” indicates no significant difference. Functional guilds are sorted in descending order of relative abundance in the control group.

## Data Availability

The original contributions presented in this study are included in the article/Supplementary Materials. Further inquiries can be directed to the corresponding authors.

## Supplementary Materials

The following supporting information can be downloaded at: https://www.mdpi.com/article/10.3390/microorganisms14050982/s1, Figure S1: Rarefaction curves of 16s rRNA,18S rRNA and ITS amplicon sequencing; Table S1: Summary of 16S rRNA, 18S rRNA and ITS amplicon sequencing read counts for each sample after each processing step; Table S2: Predicted bacterial phenotypes based on BugBase analysis of 16S rRNA gene amplicon sequencing data; Table S3: Complete list of predicted functional abundances based on FAPROTAX annotation for all 64 functional groups.
