# Supplementary material for: Amplicon Sequencing Reveals Microbial Community Structure and Its Relationships with Environmental Factors in Macrobrachium nipponense Aquaculture Ponds
Source: Microorganisms. 2026 Apr 27;14(5):982. doi: 10.3390/microorganisms14050982 (PMC13210150; doi:10.3390/microorganisms14050982)
Supplement: Supplementary file 1 [file microorganisms-14-00982-s001.zip › microorganisms-4227259-supplementary.pdf]

Amplicon Sequencing Reveals Microbial Community Structure and Its Relationships with Environmental Factors in *Macrobrachium nipponense* Aquaculture Ponds

Wanqi Zhang[a],Xiaofan Fang[b],Yuefan Zhang[b],Yiwei Xiong[c],Wenyi Zhang[ac], Shubo Jin[abc] , and Hongtuo Fu[ac],Sufei Jiang[abc*],Hui Qiao[abc*]

a Wuxi Fisheries College, Nanjing Agricultural University, Wuxi 214081, China

b Key Laboratory of Mariculture & Stock Enhancement in North China' s Sea, Ministry of Agriculture and Rural Affairs, Dalian Ocean University, Dalian 116023, China

c Key Laboratory of Freshwater Fisheries and Germplasm Resources Utilization, Ministry of Agriculture and Rural Affairs, Freshwater Fisheries Research Center, Chinese Academy of Fishery Sciences, Wuxi 214081, China

** Corresponding author : qiaoh@ffrc.cn(H.Q.), Tel.: +86-510-8555-0495, Fax:0510-8555-3304 ;*

jiangsf@ffrc.cn (S.J.),Tel.: +86-510-8745-6886, Fax:0510-8555-3304

Content: Supplementary data includes 9 figures and 5 tables in Page S2-S15:

Fig. S1. **Page S3**
Table S1. **Page S2**
Table S2. **Page S3**
Table S3. **Page S4**

Table S1. Summary of 16S rRNA, 18S rRNA and ITS amplicon sequencing read counts for each sample after each processing step.

	Sample ID	Input	Filtered	Denoised	Merged	Non-chimeric	Non-singleton
16S rRNA	L1	99879	94061	91322	83397	81594	81506
	L2	89275	84475	82017	74553	73174	73091
	L3	85236	80652	78442	71283	70094	70032
	H1	83777	78358	76160	69538	68395	68337
	H2	93449	87922	85735	78264	76481	76431
	H3	76978	72421	70661	65556	63875	63836
	O1	71045	66648	62829	55886	53943	53792
	O2	71818	67620	64147	58196	56532	56383
	O3	70511	66165	63285	56981	54373	54203
18S rRNA	L1	94924	87392	86450	81128	76900	76895
	L2	90086	83592	82700	77806	75298	75297
	L3	86789	80848	80115	76120	73537	73534
	H1	93226	86596	85575	79494	77589	77585
	H2	87470	81002	80274	75156	73354	73351
	H3	92691	85915	85119	80164	77686	77681
	O1	78923	70825	69492	65133	64135	64133
	O2	74727	67318	66061	61504	58616	58614
	O3	78854	71013	69622	64777	62056	62055
ITS	L1	92488	83707	82506	72857	72632	72632
	L2	88982	81711	80599	73773	73240	73240
	L3	78910	72134	71104	63124	62383	62383
	H1	98491	88718	87266	77674	77524	77524
	H2	96552	81961	80592	72314	72173	72173
	H3	93236	79341	78014	67843	67543	67542
	O1	94652	83415	80719	70917	70899	70895
	O2	91838	80322	77871	70171	70058	70057
	O3	89625	80723	78120	69320	69039	69038

Note: The table presents the number of sequences retained at each step of the DADA2 denoising pipeline for ITS amplicon sequencing.

Sample ID: Sample identifier. Samples L1–L3: low-nitrogen ponds; H1–H3: high-nitrogen ponds; O1–O3: external river samples (pre-culture baseline).
Input: Number of raw paired-end reads after primer trimming and before quality filtering (i.e., reads entering DADA2).
Filtered: Number of reads retained after quality filtering (DADA2 filterAndTrim step).
Denoised: Number of reads after denoising (error correction).
Merged: Number of reads after merging paired-end reads.
Non-chimeric: Number of reads after removal of chimeric sequences.

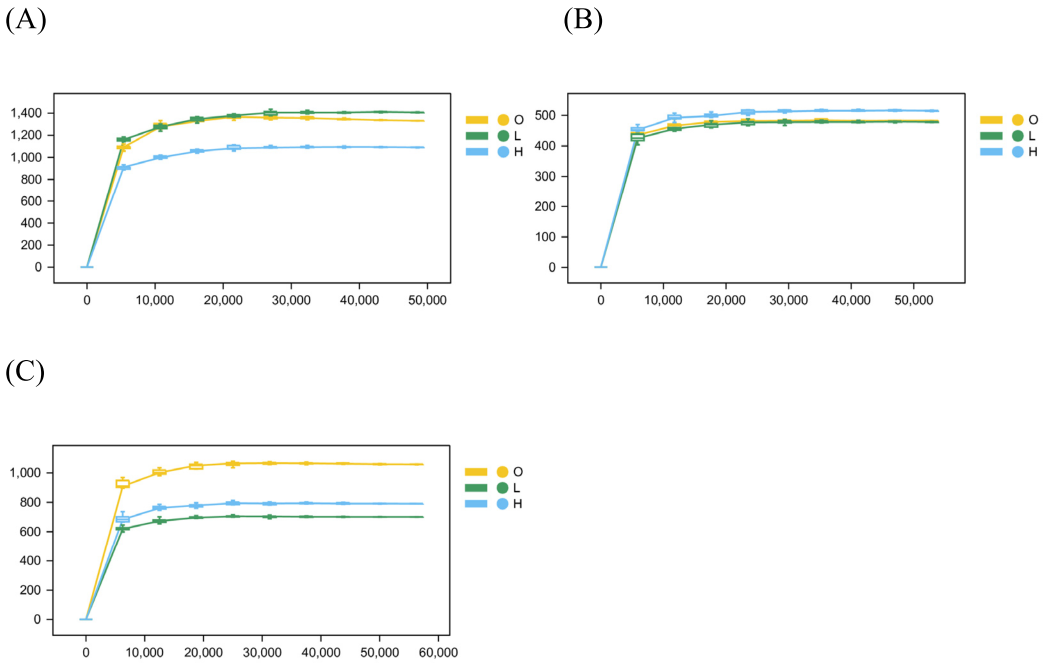

Figure S1. Rarefaction curves of 16s rRNA,18S rRNA and ITS amplicon sequencing.

Rarefaction curves showing the relationship between sequencing depth (number of reads) and observed species (ASVs) for each sample. The x-axis represents the number of reads sampled, and the y-axis represents the number of observed ASVs. The curves for all samples plateau when the sequencing depth exceeds approximately 20,000 reads, indicating that the sequencing depth was sufficient to capture the majority of fungal community diversity in the samples. Samples L1 – L3: low-nitrogen ponds; H1 – H3: high-nitrogen ponds; O1 – O3: external river samples (pre-culture baseline).

Table S2. Predicted bacterial phenotypes based on BugBase analysis of 16S rRNA gene amplicon sequencing data.

phynotype	L	H	O	ANOVA P
Potentially_Pathogenic	0.2083±0.0062^{ab}	0.2311 ± 0.0100^{b}	0.1639 ± 0.0327^{a}	0.017
Gram_Negative	0.1748 ± 0.0136^{ns}	0.2038 ± 0.0198^{ns}	0.1746 ± 0.0425^{ns}	0.401
Stress_Tolerant	0.1835 ± 0.0042^{b}	0.1538 ± 0.0077^{a}	0.1927 ± 0.0098^{b}	0.002
Forms_Biofilms	0.1004 ± 0.0135^{a}	0.0971 ± 0.0021^{a}	0.1410 ± 0.0217^{b}	0.020
Aerobic	0.0761 ± 0.0106^{ns}	0.0870 ± 0.0018^{ns}	0.1165 ± 0.0221^{ns}	0.031
Contains_Mobile_Elements	0.0749 ± 0.0054^{ns}	0.0816 ± 0.0037^{ns}	0.0898 ± 0.0318^{ns}	0.644

Gram_Positive	0.0772 ± 0.0128[ns]	0.0746 ± 0.0139[ns]	0.0625 ± 0.0269[ns]	0.624
Anaerobic	0.0615 ± 0.0163[b]	0.0398 ± 0.0039[ab]	0.0289 ± 0.0146[a]	0.052
Facultatively_Anaerobic	0.0433 ± 0.0019[a]	0.0312 ± 0.0012[b]	0.0301 ± 0.0082[ab]	0.029

Note: Values are presented as mean ± standard deviation (n = 3 per group). Groups L, H, and O represent low-nitrogen ponds, high-nitrogen ponds, and pre-culture baseline (external river), respectively. Bacterial phenotypes were predicted using BugBase based on 16S rRNA gene amplicon sequencing data. One-way analysis of variance (ANOVA) was used to compare phenotype abundances among the three groups; the *p values* from ANOVA are shown in the last column. For phenotypes with homogeneous variances (Levene's test, $P \geq 0.05$), Tukey HSD post hoc test was applied; for those with heterogeneous variances ($P < 0.05$), the Games-Howell post hoc test was applied. Different superscript letters within the same row indicate significant differences between groups based on the post hoc tests ($P < 0.05$); "ns" indicates no significant differences among groups.

Table S3. Complete list of predicted functional abundances based on FAPROTAX annotation for all 64 functional groups

FUNCTION	L	H	O	ANOVA P
methanotrophy	0.67 ± 1.15[a]	0.00 ± 0.00[a]	4082.67 ± 587.08[b]	<0.001
methylotrophy	755.33 ± 200.12[a]	408.67 ± 103.16[a]	5444.33 ± 809.04[b]	<0.001
nitrogen_fixation	70.00 ± 26.76[a]	20.67 ± 20.21[a]	2462.33 ± 556.12[b]	<0.001
photoheterotrophy	1045.00 ± 242.52[b]	278.00 ± 19.16[b]	25.67 ± 7.09[a]	<0.001
ureolysis	723.00 ± 100.21[b]	1624.00 ± 37.04[c]	236.00 ± 70.57[a]	<0.001
oxygenic_photoautotrophy	9804.33 ± 1410.00[a]	17667.67 ± 1629.90[b]	5245.00 ± 2442.11[a]	<0.001
photoautotrophy	9814.67 ± 1410.11[a]	17685.67 ± 1631.11[b]	5251.00 ± 2441.50[a]	<0.001
photosynthetic_cyanobacteria	9804.33 ± 1409.99[a]	17667.67 ± 1629.90[b]	5245.00 ± 2442.11[a]	<0.001
phototrophy	10849.33 ± 1591.11[b]	17945.67 ± 1640.11[c]	5273.00 ± 2441.11[a]	<0.001
hydrocarbon_degradation	0.67 ± 1.15[a]	0.00 ± 0.00[a]	4087.00 ± 589.11[b]	<0.001
aerobic_chemoheterotrophy	5419.33 ± 1139.61[b]	6075.00 ± 337.12[b]	2350.00 ± 263.25[a]	0.001
chlorate_reducers	10.67 ± 11.02[a]	13.00 ± 19.92[a]	151.67 ± 47.00[b]	0.002
dark_oxidation_of_sulfur_compounds	10.33 ± 5.51[a]	4.00 ± 4.58[a]	42.33 ± 11.02[b]	0.002
dark_sulfide_oxidation	8.33 ± 4.93[ns]	0.00 ± 0.00[ns]	39.00 ± 14.11[ns]	0.003
chemoheterotrophy	14352.00 ± 1019.38[ab]	11690.00 ± 523.70[b]	9025.67 ± 1535.66[a]	0.003

methanol_oxidation	754.67 ± 199.89[a]	408.67 ± 103.16[a]	1361.67 ± 247.11[b]	0.003
nitrate_respiration	35.33 ± 19.16[ns]	57.33 ± 4.16[ns]	217.67 ± 60.11[ns]	0.003
nitrogen_respiration	35.33 ± 19.16[ns]	57.33 ± 4.16[ns]	217.67 ± 60.11[ns]	0.003
fermentation	8156.00 ± 2228.71[b]	5226.00 ± 253.35[b]	1111.67 ± 965.26[a]	0.003
aerobic_ammonia_oxidation	6.00 ± 5.29[a]	94.33 ± 35.33[b]	85.67 ± 17.90[b]	0.006
nitrification	6.00 ± 5.29[a]	94.33 ± 35.33[b]	85.67 ± 17.90[b]	0.006
nonphotosynthetic_cyanobacteria	88.33 ± 23.76[b]	50.00 ± 6.00[ab]	17.67 ± 17.33[a]	0.007
human_pathogens_pneumonia	3030.33 ± 718.29[ns]	1939.00 ± 268.74[ns]	2970.67 ± 528.53[ns]	0.082
xylanolysis	215.00 ± 98.11[ns]	190.33 ± 150.31[ns]	4.33 ± 7.51[ns]	0.091
animal_parasites_or_symbionts	4710.33 ± 650.74[ns]	4165.67 ± 351.51[ns]	3515.00 ± 590.60[ns]	0.094
dark_hydrogen_oxidation	83.67 ± 33.62[ns]	33.00 ± 17.44[ns]	41.33 ± 23.88[ns]	0.107
iron_respiration	0.00 ± 0.00[ns]	0.00 ± 0.00[ns]	19.33 ± 10.41[ns]	0.11
anoxygenic_photoautotrophy_S_oxidizing	10.33 ± 5.77[ns]	18.00 ± 5.57[ns]	6.00 ± 7.21[ns]	0.134
anoxygenic_photoautotrophy	10.33 ± 5.77[ns]	18.00 ± 5.57[ns]	6.00 ± 7.21[ns]	0.134
chloroplasts	3835.00 ± 732.99[ns]	1521.33 ± 179.92[ns]	4750.00 ± 2863.54[ns]	0.135
nitrate_reduction	429.00 ± 160.22[ns]	211.33 ± 72.72[ns]	484.33 ± 198.63[ns]	0.149
intracellular_parasites	433.33 ± 89.76[b]	296.33 ± 15.50[ab]	222.33 ± 67.24[a]	0.2
cellulolysis	28.33 ± 4.16[b]	19.00 ± 28.51[ab]	1.67 ± 1.53a	0.267
aromatic_compound_degradation	21.00 ± 5.20[ns]	20.33 ± 27.06[ns]	180.00 ± 223.16[ns]	0.295
manganese_oxidation	8.67 ± 7.51[ns]	29.33 ± 26.08[ns]	70.00 ± 74.32[ns]	0.317
respiration_of_sulfur_compounds	31.67 ± 29.94[ns]	72.33 ± 29.69[ns]	85.33 ± 61.65[ns]	0.348

sulfate_respiration	31.67 ± 29.94^{ns}	72.33 ± 29.69^{ns}	85.33 ± 61.65^{ns}	0.348
sulfite_respiration	1.00 ± 1.73^{ns}	0.00 ± 0.00^{ns}	13.67 ± 20.26^{ns}	0.35
dark_sulfur_oxidation	0.00 ± 0.00^{ns}	0.00 ± 0.00^{ns}	0.67 ± 1.15^{ns}	0.422
fumarate_respiration	0.00 ± 0.00^{ns}	0.00 ± 0.00^{ns}	0.67 ± 1.15^{ns}	0.422
anoxygenic_photoautotrophy_H2_oxidizing	0.00 ± 0.00^{ns}	0.00 ± 0.00^{ns}	3.67 ± 6.35^{ns}	0.422
arsenate_respiration	0.00 ± 0.00^{ns}	0.00 ± 0.00^{ns}	10.67 ± 18.48^{ns}	0.422
dark_iron_oxidation	0.00 ± 0.00^{ns}	0.00 ± 0.00^{ns}	3.67 ± 6.35^{ns}	0.422
dark_thiosulfate_oxidation	0.00 ± 0.00^{ns}	0.00 ± 0.00^{ns}	0.67 ± 1.15^{ns}	0.422
nitrate_ammonification	0.00 ± 0.00^{ns}	0.00 ± 0.00^{ns}	11.33 ± 19.63^{ns}	0.422
nitrite_ammonification	0.00 ± 0.00^{ns}	0.00 ± 0.00^{ns}	11.33 ± 19.63^{ns}	0.422
plastic_degradation	0.00 ± 0.00^{ns}	0.00 ± 0.00^{ns}	13.33 ± 23.09^{ns}	0.422
thiosulfate_respiration	0.00 ± 0.00^{ns}	0.00 ± 0.00^{ns}	0.67 ± 1.15^{ns}	0.422
dissimilatory_arsenate_reduction	0.00 ± 0.00^{ns}	0.00 ± 0.00^{ns}	10.67 ± 18.48^{ns}	0.422
human_pathogens_gastroenteritis	0.00 ± 0.00^{ns}	0.00 ± 0.00^{ns}	3.00 ± 5.20^{ns}	0.422
human_associated	4105.00 ± 699.16^{ns}	3863.33 ± 215.07^{ns}	3504.33 ± 585.01^{ns}	0.442
nitrite_respiration	5.00 ± 8.66^{ns}	0.00 ± 0.00^{ns}	15.00 ± 23.43^{ns}	0.476
plant_pathogen	5.00 ± 8.66^{ns}	0.00 ± 0.00^{ns}	4.67 ± 4.51^{ns}	0.517
denitrification	5.00 ± 8.66^{ns}	0.00 ± 0.00^{ns}	1.67 ± 2.89^{ns}	0.533
nitrate_denitrification	5.00 ± 8.66^{ns}	0.00 ± 0.00^{ns}	1.67 ± 2.89^{ns}	0.533
nitrite_denitrification	5.00 ± 8.66^{ns}	0.00 ± 0.00^{ns}	1.67 ± 2.89^{ns}	0.533

nitrous_oxide_denitrification	5.00 ± 8.66^{ns}	0.00 ± 0.00^{ns}	1.67 ± 2.89^{ns}	0.533
aerobic_anoxygenic_phototrophy	1.33 ± 2.31^{ns}	0.00 ± 0.00^{ns}	3.67 ± 6.35^{ns}	0.542
human_pathogens_all	3713.33 ± 813.01^{ns}	3593.00 ± 244.51^{ns}	3252.33 ± 467.52^{ns}	0.605
human_pathogens_nosocomia	0.00 ± 0.00^{ns}	9.67 ± 16.77^{ns}	13.33 ± 23.09^{ns}	0.617
chitinolysis	1.00 ± 1.73^{ns}	0.00 ± 0.00^{ns}	13.00 ± 10.58^{ns}	0.76
human_gut	391.67 ± 188.13^{ns}	270.33 ± 73.17^{ns}	260.00 ± 370.70^{ns}	0.772
mammal_gut	391.67 ± 188.13^{ns}	270.33 ± 73.17^{ns}	260.00 ± 370.70^{ns}	0.772
predatory_or_exoparasitic	343.33 ± 81.66^{ns}	355.67 ± 46.06^{ns}	302.67 ± 232.52^{ns}	0.897

Groups L, H, and O represent low-nitrogen ponds, high-nitrogen ponds, and the pre-culture baseline (external river), respectively. Functional abundances were predicted using FAPROTAX based on 16S rRNA gene amplicon sequencing data. Values are presented as mean ± standard deviation (n = 3 per group). One-way analysis of variance (ANOVA) was used for overall comparisons among the three groups. For functions with homogeneous variances (Levene's test, $P \geq 0.05$), Tukey's HSD post hoc test was applied; for those with heterogeneous variances ($P < 0.05$), the Games-Howell post hoc test was applied. Different superscript letters (a, b, c) within the same row indicate statistically significant differences between groups ($P < 0.05$); "ns" indicates that although the overall ANOVA $P < 0.05$, no pairwise comparison reached statistical significance. Functions with ANOVA $P \geq 0.05$ are listed without superscript letters, indicating no significant differences among groups.
